# Association of Complete Blood Count Parameters and Systemic Inflammatory Indices with Early Surfactant Administration in Preterm Infants

**DOI:** 10.3390/diagnostics16203394

**Published:** 2026-10-19

**Authors:** Saime Sundus Uygun, Nihal Comez, Osman Selcuk Duysak, Evrim Kilicli, Murat Konak, Hanifi Soylu

**Affiliations:** 1 Division of Neonatology, Department of Pediatrics, Selçuk University, 42130 Konya, Türkiyedrevrimkilicli@gmail.com (E.K.); drmkonak@hotmail.com (M.K.); 2 Department of Pediatrics, Selçuk University, 42130 Konya, Türkiye; 3 Division of Neonatology, Department of Pediatrics, University of Manitoba, Winnipeg, MB R3T 2N2, Canada

**Keywords:** complete blood count, systemic inflammatory indices, preterm infants, respiratory distress syndrome, surfactant therapy

## Abstract

**Background/Objectives**: The decision to administer surfactant therapy to preterm newborns is typically based on clinical respiratory findings, oxygen requirements, and the level of respiratory support. The aim of this study was to assess the association of admission complete blood count parameters and derived systemic inflammatory indices with surfactant administration within the first 24 h of life in preterm infants born at ≤32 weeks of gestation, and to evaluate their discriminative performance. **Methods:** This single-center, retrospective observational cohort study included 100 preterm infants with a gestational age of ≤32 weeks who were admitted to the neonatal intensive care unit between 1 January 2025 and 31 March 2026. The results of the initial complete blood count and blood gas analysis, obtained as part of routine clinical care at admission, were recorded. Systemic inflammatory indices, including the neutrophil-to-lymphocyte ratio (NLR), platelet-to-lymphocyte ratio (PLR), monocyte-to-lymphocyte ratio (MLR), red cell distribution width-to-platelet ratio (RPR), systemic immune-inflammation index (SII), and mean platelet volume-to-platelet ratio (MPV/PLT), were calculated from the complete blood count results. The primary outcome was surfactant administration within the first 24 h of life. Associated factors were evaluated using logistic regression analysis, and the discriminative performance of continuous variables was assessed using receiver operating characteristic (ROC) curve analysis. **Results:** Surfactant was administered to 45 infants (45%) within the first 24 h of life. Infants who received surfactant had lower gestational ages, birth weights, 1- and 5 min Apgar scores, partial pressure of carbon dioxide (PCO_2_), hemoglobin levels, and hematocrit values. No significant differences were observed between groups in NLR, PLR, MLR, RPR, SII, or MPV/PLT. In multivariable analysis, lower gestational age (OR = 0.65; 95% CI: 0.50–0.84; *p* = 0.001), small-for-gestational-age (SGA) status (OR = 4.61; 95% CI: 1.25–16.91; *p* = 0.021), and lower 1 min Apgar score (OR = 0.67; 95% CI: 0.46–0.96; *p* = 0.031) were independently associated with early surfactant administration. Hemoglobin was not independently associated with early surfactant administration after adjustment. In the ROC analysis, the AUC values were 0.804 for birth weight, 0.776 for gestational age, 0.694 for hemoglobin, 0.677 for hematocrit, and 0.640 for PCO_2_. **Conclusions:** Lower gestational age, SGA status, and lower 1 min Apgar score were independently associated with early surfactant administration. No significant associations were observed between the evaluated complete blood count-derived inflammatory indices and early surfactant administration. Although hemoglobin and hematocrit levels differed between groups, hemoglobin was not independently associated with the outcome after adjustment. ROC analysis provided descriptive estimates of the discriminative performance of gestational age, birth weight, hemoglobin, hematocrit, and PCO_2_.

## 1. Background/Objectives

Preterm birth remains one of the leading causes of neonatal mortality and morbidity. Structural and functional immaturity of the lungs and insufficient pulmonary surfactant production play key roles in the development of respiratory distress syndrome (RDS) in preterm infants [1]. The incidence and severity of RDS are inversely related to gestational age; therefore, infants born at very low gestational ages more frequently require respiratory support and surfactant therapy [2].

The management of neonatal respiratory distress syndrome (RDS) involves appropriate stabilization at birth, early noninvasive respiratory support, and timely surfactant administration when indicated [3]. According to the European Consensus Guidelines on the Management of Respiratory Distress Syndrome: 2025, decisions regarding surfactant treatment should consider the progression of respiratory distress, oxygen requirements, and the level of respiratory support [2]. Clinical signs, including increased work of breathing and chest retractions, are evaluated alongside the fraction of inspired oxygen (FiO_2_) and the degree of lung aeration assessed by chest radiography or lung ultrasound. For infants born after 28 weeks of gestation with worsening RDS, the guidelines suggest surfactant administration during noninvasive respiratory support when the mean airway pressure is ≥6 cmH_2_O, the FiO_2_ requirement is ≥0.30, or lung ultrasound findings indicate surfactant need. In preterm infants requiring endotracheal intubation for stabilization, surfactant should be administered as soon as possible. Radiographic findings may support the diagnosis of RDS, but treatment decisions should not rely on chest radiography alone. In this context, investigating the associations of readily available, low-cost laboratory parameters with early surfactant administration may provide additional insight into the clinical characteristics of affected preterm infants.

Complete blood count and blood gas analysis are routinely performed during the early postnatal period in neonatal intensive care units (NICUs) [4]. Blood gas parameters provide information about the infant’s respiratory and metabolic status, whereas complete blood count parameters may reflect hematological and inflammatory responses [5,6]. Leukocyte, neutrophil, lymphocyte, monocyte, and platelet counts, as well as hemoglobin, hematocrit, red cell distribution width (RDW), and mean platelet volume (MPV), may be influenced by gestational age, postnatal age, fetal growth, perinatal stress, and inflammatory processes. Therefore, developmental and perinatal characteristics should be considered when interpreting complete blood count results in preterm infants [7].

Routine complete blood count parameters may be influenced by gestational age and postnatal adaptation, as well as by perinatal stress and inflammatory processes [7]. Composite indices derived from complete blood count parameters, including the neutrophil-to-lymphocyte ratio, platelet-to-lymphocyte ratio, monocyte-to-lymphocyte ratio, red cell distribution width-to-platelet ratio, mean platelet volume-to-platelet ratio, and systemic immune-inflammation index, are readily available markers that may reflect the systemic inflammatory response. The associations of these parameters measured early in life with early surfactant administration, as well as their discriminative performance, remain unclear in preterm infants.

This study aimed to evaluate the association of complete blood count parameters obtained at NICU admission and complete blood count-derived inflammatory indices with early surfactant administration within the first 24 h of life in preterm infants born at ≤32 weeks of gestation.

## 2. Methods

### 2.1. Study Design and Setting

This retrospective, single-center observational cohort study was conducted in the neonatal intensive care unit (NICU) of Selçuk University Faculty of Medicine, a tertiary-level referral center in Konya, Türkiye. The study included preterm infants born at ≤32 weeks of gestation who were admitted to the NICU between 1 January 2025 and 31 March 2026. During the study period, respiratory management was based on clinical assessment of respiratory distress, oxygen requirements, and the level of respiratory support. Noninvasive respiratory support was used when clinically appropriate, while endotracheal intubation and invasive mechanical ventilation were provided when required. The institutional criteria for surfactant administration are described in Section 2.3.

### 2.2. Study Population and Patient Selection

A total of 127 preterm infants born at ≤32 weeks of gestation were assessed for eligibility.

The inclusion criteria:

- Gestational age of ≤32 weeks;
- Admission to the neonatal intensive care unit during the study period;
- Availability of complete blood count results obtained at admission;
- Availability of information on surfactant administration within the first 24 h of life
- Availability of the clinical and laboratory data required for the study.

The exclusion criteria:

- Presence of a major structural anomaly diagnosed during the antenatal or postnatal period;
- Presence of congenital heart disease;

Based on these criteria, 27 patients were excluded from the study, and a total of 100 patients were included in the final analysis.

### 2.3. Surfactant Administration

Surfactant administration during the study period was based on the clinical diagnosis of respiratory distress syndrome (RDS), considering the severity of respiratory distress, oxygen requirements, and the level of respiratory support. Chest radiographic findings were used to support the diagnosis of RDS when available. In infants receiving different noninvasive ventilation (NIV) modes, surfactant was administered when the fraction of inspired oxygen (FiO_2_) exceeded 0.35 despite a continuous positive airway pressure (CPAP) or positive end-expiratory pressure (PEEP) level of ≥7 cmH_2_O. No fixed duration of sustained FiO_2_ elevation was required before surfactant administration; treatment decisions were based on the persistence or progression of respiratory distress and oxygen requirements despite appropriate noninvasive respiratory support. Infants requiring endotracheal intubation for stabilization could also receive surfactant. Treatment decisions were based on the overall clinical assessment rather than radiographic findings alone. The institutional surfactant administration criteria remained unchanged throughout the study period.

Patients were categorized according to whether they received surfactant within the first 24 h of life. The early surfactant administration group comprised 45 infants, whereas the group without early surfactant administration comprised 55 infants. Of the 45 infants who received surfactant, six were treated in the delivery room before NICU admission and collection of the first complete blood count sample. These infants were included in the early surfactant administration group because treatment was administered within the first 24 h of life.

### 2.4. Data Collection and Study Variables

The patients’ demographic, perinatal, clinical, and laboratory data were obtained retrospectively from the hospital’s electronic record system and patient files. In terms of demographic and perinatal variables, gestational age, birth weight, sex, birth weight classification by gestational week (SGA, AGA, or LGA), mode of delivery, first- and fifth-minute Apgar scores, administration of antenatal steroids, and respiratory support provided in the delivery room were recorded. Respiratory support in the delivery room was assessed according to the use of nasal continuous positive airway pressure (CPAP) or endotracheal intubation. Surfactant administration within the first 24 h of life was recorded as the primary outcome.

Gestational age was calculated based on the first day of the last menstrual period. Birth weight was classified according to the Fenton preterm growth charts using gestational age- and sex-specific percentiles. Infants with birth weights below the 10th percentile were classified as small for gestational age (SGA), those between the 10th and 90th percentiles as appropriate for gestational age (AGA), and those above the 90th percentile as large for gestational age (LGA) [8].

For the laboratory evaluation, the first complete blood count (CBC) and blood gas results obtained as part of routine clinical care at NICU admission were used. No additional tests were performed for the study. CBC analyses were performed using venous blood samples on a CELL-DYN 3200 hematology analyzer (Abbott Diagnostics, Abbott Park, IL, USA). Venous blood gas analyses were performed using blood samples obtained from either a peripheral vein or the umbilical vein and analyzed with a RAPIDPoint 500 blood gas analyzer (Siemens Healthcare Diagnostics Inc., Tarrytown, NY, USA)

The CBC parameters evaluated included white blood cell count (WBC), hemoglobin (HGB), hematocrit (HCT), platelet count (PLT), neutrophil, lymphocyte, and monocyte counts, mean platelet volume (MPV), and red cell distribution width (RDW). The blood gas parameters pH and partial pressure of carbon dioxide (PCO_2_) were recorded.

The exact intervals between birth and CBC or blood gas sampling, as well as the type and level of respiratory support at the time of sampling, were not systematically recorded in the retrospective dataset.

- Neutrophil-to-lymphocyte ratio (NLR) = neutrophil count/lymphocyte count
- Monocyte-to-lymphocyte ratio (MLR) = monocyte count/lymphocyte count
- Platelet-to-lymphocyte ratio (PLR) = platelet count/lymphocyte count
- Red cell distribution width (RDW)-to-platelet ratio (RPR) = RDW/platelet count
- Systemic immune-inflammation index (SII) = platelet count × neutrophil count/lymphocyte count
- Mean platelet volume-to-platelet ratio (MPV/PLT) = MPV/platelet count

The primary outcome was early surfactant administration, defined as treatment within the first 24 h of life. Its associations with complete blood count parameters and derived systemic inflammatory indices were evaluated.

The diagnosis of RDS was made based on a combined evaluation of clinical findings and chest X-ray findings. Surfactant therapy was administered when oxygen requirements remained above 35% despite a PEEP level of ≥7 cmH_2_O under CPAP. Surfactant was also administered to infants who required endotracheal intubation for respiratory stabilization.

### 2.5. Statistical Analysis

Statistical analyses were performed using IBM SPSS Statistics for Windows, IBM SPSS Statistics, version 25.0 (IBM Corp., Armonk, NY, USA)

The normality of continuous variables was assessed using visual methods and the Shapiro–Wilk test. Normally distributed continuous variables are presented as mean ± standard deviation, whereas non-normally distributed variables are presented as median (interquartile range). Categorical variables are expressed as numbers and percentages.

Infants who received surfactant therapy within the first 24 h of life were compared with those who did not. Depending on the distribution of the data, continuous variables were compared using the independent-samples *t*-test or Mann–Whitney *U* test. Categorical variables were compared using Pearson’s chi-square test or Fisher’s exact test, as appropriate.

Associations between clinical and laboratory variables and early surfactant administration were evaluated using binary logistic regression analysis. Variables with a *p*-value < 0.20 in univariable logistic regression analyses were considered candidates for multivariable analysis, together with clinically relevant perinatal factors. To reduce redundancy among correlated variables, gestational age was included instead of birth weight, the 1 min Apgar score instead of the 5 min Apgar score, and hemoglobin instead of hematocrit. The final multivariable model included gestational age, small-for-gestational-age (SGA) status, the 1 min Apgar score, and hemoglobin. Multicollinearity was assessed by examining correlations among candidate variables and calculating variance inflation factors (VIFs) for the variables included in the final model. The results are presented as odds ratios (ORs) with 95% confidence intervals (CIs).

Given the potential clinical overlap between delivery-room intubation and early surfactant administration, delivery-room intubation was not included in the primary multivariable model. An additional sensitivity analysis was performed by including delivery-room intubation as a covariate. This analysis was restricted to infants with complete data for all included variables.

To address the temporal relationship between blood sampling and surfactant administration, a separate sensitivity analysis was performed after excluding the six infants who received surfactant before the first CBC sample was obtained. In the remaining infants who received surfactant, CBC sampling preceded surfactant administration.

The discriminative performance of continuous variables significantly associated with early surfactant administration in univariable analyses was evaluated using receiver operating characteristic (ROC) curve analysis. The area under the curve (AUC) and its 95% confidence interval were calculated. Optimal cutoff values were determined using the Youden index, and the corresponding sensitivity and specificity values were reported.

### 2.6. Ethical Approval

This study was approved by the Local Ethics Committee of the Dean’s Office of Selçuk University Faculty of Medicine (meeting date: 21 April 2026; decision no. 2026/275). Written informed consent was obtained from the parents or legal guardians of the infants included in the study. The study was conducted in accordance with the principles of the Declaration of Helsinki, and all patient data were de-identified before analysis.

## 3. Results

A total of 100 preterm newborns were included in the study. Surfactant therapy was administered to 45 of the patients (45%) within the first 24 h of life, while 55 (55%) did not receive surfactant. The gestational ages and birth weights of patients who received surfactant were significantly lower than those of patients who did not receive surfactant (*p* < 0.001 for both). The Apgar scores at 1 and 5 min were also significantly lower in the group that received surfactant (*p* < 0.001 for both).

Information on delivery-room respiratory support was unavailable for six infants (five in the early surfactant administration group and one in the non-surfactant group) because the relevant medical records could not be retrieved or the infants had been referred from other healthcare facilities. Among infants with available data, delivery-room intubation was significantly more frequent in the early surfactant administration group than in the non-surfactant group (13/40 [32.5%] vs. 4/54 [7.4%], respectively; *p* = 0.004).

No statistically significant differences were observed between the groups in terms of sex, birth weight classification, mode of delivery, or antenatal corticosteroid administration (all *p* > 0.05).

A comparison of the patients’ demographic and perinatal characteristics by group is presented in Table 1.

Blood gas levels, complete blood counts, and inflammatory markers were compared between patients who received surfactant and those who did not. The PCO_2_ level was significantly lower in the surfactant group (*p* = 0.018). Hemoglobin and hematocrit levels were also significantly lower in the surfactant group (*p* = 0.001 and *p* = 0.002, respectively).

No significant differences were observed between the groups in terms of pH, white blood cell count, platelet count, RDW, MPV, neutrophil, lymphocyte, or monocyte counts, or systemic inflammatory indices (all *p* > 0.05).

Complete data were available for the calculation of RPR and MPV/PLT in all 100 infants. However, NLR, PLR, MLR, and SII could be calculated in only 84 infants because differential leukocyte counts were not reported in the laboratory results of 16 infants. The specific reasons for the absence of these results could not be determined retrospectively. Missing data for these indices were more frequent in the early surfactant administration group (14/45, 31.1%) than in the non-surfactant group (2/55, 3.6%).

Comparisons of laboratory parameters between the groups are presented in Table 2.

In the univariable logistic regression analysis, lower gestational age, birth weight, 1- and 5 min Apgar scores, hemoglobin levels, and hematocrit values were associated with early surfactant administration. SGA status showed a statistically significant borderline association with increased odds of early surfactant administration (*p* = 0.049).

Gestational age, SGA status, 1 min Apgar score, and hemoglobin level were included in the multivariable logistic regression model. In the multivariable model, each one-week increase in gestational age was associated with approximately a 35% reduction in the odds of early surfactant administration (OR = 0.65; 95% CI: 0.50–0.84; *p* = 0.001). Infants with SGA status had approximately 4.6-fold higher odds of early surfactant administration than non-SGA infants (OR = 4.61; 95% CI: 1.25–16.91; *p* = 0.021. Each one-point increase in the 1 min Apgar score was associated with approximately a 33% reduction in the odds of early surfactant administration (OR = 0.67; 95% CI: 0.46–0.96; *p* = 0.031).

Hemoglobin level, which was associated with early surfactant administration in the univariable analysis, was no longer independently associated with the outcome after adjustment for gestational age, SGA status, and 1 min Apgar score (OR = 0.99; 95% CI: 0.78–1.25; *p* = 0.911).

Collinearity assessment showed strong correlations between hemoglobin and hematocrit (r = 0.967), the 1 min and 5 min Apgar scores (r = 0.871), and gestational age and birth weight (r = 0.781). In the final multivariable model, VIF values ranged from 1.03 to 1.62, indicating no substantial multicollinearity among the included variables. In an additional sensitivity analysis including delivery-room intubation as a covariate (*n* = 91), gestational age remained significantly associated with early surfactant administration (OR = 0.64; *p* < 0.001). In contrast, SGA status (OR = 3.03; *p* = 0.127), the 1 min Apgar score (OR = 0.75; *p* = 0.226), and hemoglobin (OR = 0.99; *p* = 0.919) were not statistically significant.

Delivery-room intubation was not independently associated with early surfactant administration in this model (OR = 1.47; *p* = 0.680).

A separate sensitivity analysis was performed after excluding the six infants who received surfactant before the first CBC sample was obtained. The remaining cohort comprised 94 infants, including 39 who received surfactant and 55 who did not. Hemoglobin and hematocrit levels remained significantly lower in the early surfactant group (*p* = 0.003 and *p* = 0.008, respectively), whereas none of the evaluated CBC-derived inflammatory indices showed statistically significant between-group differences (all *p* > 0.05).

In the multivariable logistic regression analysis, which included 90 infants with complete data, lower gestational age (adjusted OR = 0.67; 95% CI: 0.52–0.87; *p* = 0.002), SGA status (adjusted OR = 4.39; 95% CI: 1.18–16.27; *p* = 0.027), and lower 1 min Apgar score (adjusted OR = 0.68; 95% CI: 0.47–0.99; *p* = 0.044) remained significantly associated with early surfactant administration. Hemoglobin was not independently associated with the outcome (adjusted OR = 0.98; 95% CI: 0.77–1.24; *p* = 0.848). Overall, the direction and statistical significance of these associations were consistent with those observed in the primary analysis.

The primary multivariable logistic regression model, which included gestational age, SGA status, the 1 min Apgar score, and hemoglobin, was evaluated in 96 infants with complete data. The model demonstrated an apparent AUC of 0.854 (95% bootstrap CI: 0.773–0.924). The Hosmer–Lemeshow goodness-of-fit test did not indicate a statistically significant lack of fit (χ^2^ = 8.48, df = 8, *p* = 0.388), and the Brier score was 0.157. These measures reflect model performance in the development cohort and should not be interpreted as externally validated performance.

The results of the logistic regression analysis are presented in Table 3.

Receiver operating characteristic (ROC) curve analysis was performed to evaluate the discriminative performance of gestational age, birth weight, hemoglobin, hematocrit, and PCO_2_ for early surfactant administration. The AUC values were 0.776 (95% CI: 0.683–0.870) for gestational age, 0.804 (95% CI: 0.715–0.893) for birth weight, 0.694 (95% CI: 0.589–0.799) for hemoglobin, 0.677 (95% CI: 0.570–0.784) for hematocrit, and 0.640 (95% CI: 0.528–0.751) for PCO_2_.

Optimal cutoff values were determined using the Youden index. The corresponding sensitivity and specificity estimates, together with their 95% confidence intervals, are presented in Table 4. Because these cutoff values were derived and evaluated in the same cohort, they should be considered exploratory and require external validation before clinical application.

## 4. Discussion

Prematurity and low birth weight are the principal risk factors for the development of RDS, and its incidence increases markedly with decreasing gestational age and birth weight. Consistent with this relationship, the 2026 Respiratory Distress Syndrome and Surfactant Therapy Guideline of the Turkish Neonatal Society reports, based on multicenter cohort data, that the incidence of RDS is approximately 98% among infants born at 24 weeks of gestation, decreasing to 5% at 34 weeks and to below 1% at 37 weeks [9,10]. Although our study included only infants born at ≤32 weeks of gestation and evaluated surfactant administration within the first 24 h of life rather than the development of RDS itself, our findings are consistent with the inverse relationship between gestational maturity and RDS risk emphasized in the 2026 guideline of the Turkish Neonatal Society [9]. The lower gestational age and birth weight observed among surfactant-treated infants likely reflect greater pulmonary immaturity and more pronounced endogenous surfactant deficiency. The independent association between gestational age and early surfactant administration, together with the greater discriminative performance of gestational age and birth weight compared with the evaluated laboratory parameters, further underscores the clinical importance of the degree of prematurity. These findings suggest that biological maturity remains more informative in assessing early surfactant administration than routine hematological parameters

Current approaches to early surfactant therapy recommend that treatment decisions be based primarily on oxygen requirement, the severity of respiratory distress, the level of noninvasive airway pressure, and lung imaging findings. In the latest European Consensus Guidelines on the Management of RDS, Sweet et al. noted that the available evidence regarding biochemical markers and lung function tests for predicting early surfactant administration in the early postnatal period remains insufficient to support their routine clinical use [11]. The lack of an association between complete blood count-derived inflammatory indices and early surfactant administration in our study, together with the greater discriminative performance of gestational age and birth weight compared with hematological parameters, is consistent with this perspective. These findings indicate that admission complete blood count parameters alone are insufficient to identify early surfactant administration and should not replace direct respiratory and clinical indicators in treatment decisions. Further prospective studies using standardized sampling before surfactant administration are needed to identify objective and readily available laboratory biomarkers that may complement clinical assessment and improve the early identification of infants likely to require surfactant therapy.

The effects of fetal growth restriction on RDS and early surfactant administration have not been clearly established. In a review by Malhotra et al. evaluating surfactant therapy in preterm infants with intrauterine growth restriction (IUGR), the authors reported a lack of adequate studies directly comparing preterm infants with IUGR and those who were appropriate for gestational age (AGA). In the only eligible study, small-for-gestational-age (SGA) status was used as a surrogate for IUGR, and no differences were observed between SGA and AGA infants in terms of RDS severity or the rates of intubation and surfactant administration. However, SGA infants required oxygen therapy and CPAP for longer periods and had higher rates of bronchopulmonary dysplasia and mortality [12]. In our study, the association between SGA status and early surfactant administration became statistically significant only after adjustment for gestational age, 1 min Apgar score, and hemoglobin level, suggesting that fetal growth status should be interpreted in conjunction with the degree of prematurity and the infant’s perinatal clinical condition. Unlike the previous study, we used surfactant administration within the first 24 h of life as the outcome measure; differences in patient characteristics and criteria for surfactant administration may therefore partly explain the discrepancy between the findings. Nevertheless, given the wide confidence interval around the effect estimate, our finding regarding SGA status should be interpreted cautiously and confirmed in studies with larger sample sizes. Although SGA status was independently associated with early surfactant administration in the primary multivariable model, the wide confidence interval (OR = 4.61; 95% CI: 1.25–16.91) indicates considerable uncertainty regarding the magnitude of this association. Furthermore, the association was no longer statistically significant in the sensitivity analysis that additionally included delivery-room intubation. Therefore, this finding should be interpreted cautiously and requires confirmation in larger cohorts.

In our study, a lower 1 min Apgar score remained independently associated with early surfactant administration after adjustment for gestational age, SGA status, and hemoglobin level. Although a low Apgar score is not a specific indicator of surfactant deficiency, it may reflect impaired cardiorespiratory adaptation immediately after birth and a greater need for respiratory support. The Canadian Paediatric Society guideline, based on findings reported by Brix et al., also identifies a low 5 min Apgar score as one of the factors associated with extubation failure following the INSURE procedure [13,14]. Although this evidence does not directly address the likelihood of surfactant administration, it supports a possible association between a low Apgar score and a less favorable early respiratory course in preterm infants. Nevertheless, because Apgar scores may be influenced by gestational age, delivery room resuscitation, and interobserver variability, they should not be considered a standalone criterion for determining early surfactant administration.

Evidence regarding the relationship between hemoglobin and hematocrit levels and RDS in preterm infants remains limited. In our study, admission hemoglobin and hematocrit levels were lower among infants who received surfactant. Similarly, Aslamzai et al. reported that preterm infants with RDS had lower mean hemoglobin levels than those without RDS and that neonatal anemia was independently associated with RDS, possibly through reduced oxygen-carrying capacity and consequent hypoxia [15]. However, in our study, hemoglobin was no longer independently associated with early surfactant administration after adjustment for gestational age, SGA status, and 1 min Apgar score. Although hemoglobin and hematocrit levels differed between the groups, hemoglobin was not independently associated with early surfactant administration after adjustment for gestational age, SGA status, and the 1 min Apgar score. Differences between the studies may be attributable to the use of different outcomes—RDS diagnosis versus early surfactant administration—as well as differences in the gestational age distributions of the study populations and the definitions of anemia used.

Complete blood count-derived inflammatory indices have been investigated in neonates, particularly in relation to infection and later-onset inflammation-related morbidities; however, evidence regarding their association with early surfactant administration remains limited. Regassa et al. reported higher NLR, PLR, MLR, RPR, and MPV-to-platelet ratios in infants with neonatal sepsis [16]. Similarly, another study investigating BPD in preterm infants found that PLR and RPR were associated with BPD at specific postnatal time points [17]. In our study, however, admission NLR, PLR, MLR, RPR, SII, and MPV-to-platelet ratio did not differ between infants who received early surfactant and those who did not. Previous studies focused on outcomes with prominent inflammatory components, such as sepsis and BPD, whereas our study evaluated an early clinical outcome primarily related to pulmonary immaturity and surfactant deficiency. This difference in the underlying pathophysiology and timing of the evaluated outcomes may partly explain the discrepant findings.

Studies focusing more specifically on neonatal RDS have reported conflicting findings. In a cohort of 931 preterm infants born at ≤32 weeks of gestation, Çakır et al. found higher NLR, PIV, and SII values within the first hour of life among infants with RDS and demonstrated that an SII value ≥ 78.2 was independently associated with RDS [18]. In contrast, Akın et al., in a study of 365 preterm infants born at <32 weeks of gestation, reported lower MLR, NLR, PIV, SII, and SIRI values in the RDS group. In that study, an SII value ≥ 239 was associated with a lower risk of RDS in the overall cohort, while higher SII, PIV, and SIRI values were associated with a reduced risk of RDS among infants born at ≤28 weeks of gestation [19]. Our finding that complete blood count-derived inflammatory indices were not associated with early surfactant administration differs from both patterns. The inconsistent results across studies may reflect the complex and potentially bidirectional effects of perinatal inflammation on fetal lung maturation, as well as differences in gestational age distributions, antenatal conditions, timing of blood sampling, and outcome definitions. In particular, whereas the previous studies evaluated the diagnosis of RDS, our study used surfactant administration within the first 24 h of life as the outcome; therefore, these indices may not reflect early treatment requirement in the same manner as they reflect the presence of RDS.

Blood gas analysis provides objective information regarding respiratory and ventilatory status and contributes to the clinical assessment of preterm infants with RDS. In a study of 405 preterm infants born at ≤32 weeks of gestation, Kör and Çakır reported lower admission PCO_2_ values among infants with RDS who required surfactant than among those who did not. The authors attributed this finding to the more intensive respiratory support and mechanical ventilation provided to infants in the surfactant-treated group [20].

In the present study, PCO_2_ levels were significantly lower among infants who received early surfactant administration. However, this finding should be interpreted cautiously because blood gas values may be influenced by the timing of sampling, the type of blood gas sample, and the respiratory support provided before sampling. As these factors were not standardized in our retrospective cohort, the observed difference in PCO_2_ cannot be attributed solely to differences in the severity of respiratory distress.

ROC analysis yielded AUC values of 0.804 for birth weight, 0.776 for gestational age, 0.694 for hemoglobin, 0.677 for hematocrit, and 0.640 for PCO_2_. Although the numerical AUC values differed, formal pairwise comparisons between ROC curves were not performed. Therefore, these findings should be regarded as descriptive estimates of discriminative performance rather than evidence that gestational age or birth weight provides superior clinical discrimination compared with the evaluated laboratory parameters. Furthermore, the retrospective design, potential differences in sampling timing, and the influence of respiratory management limit the clinical interpretation of these findings.

One of the main strengths of our study is that the analyses were based on the first complete blood count obtained at NICU admission as part of routine clinical care. No additional blood samples were collected for research purposes, supporting the practical applicability of this approach while avoiding additional blood loss in preterm infants with limited circulating blood volume. Complete blood count is an inexpensive, readily available laboratory test routinely performed in most NICUs. In addition to conventional hematological parameters, we simultaneously evaluated several indices—including NLR, PLR, MLR, RPR, SII, and the MPV-to-platelet ratio—that could be calculated from the same results without additional sampling or cost, thereby enabling a more comprehensive assessment of the information provided by the complete blood count. Although the diagnosis of RDS and the decision to administer surfactant are generally based on gestational age and direct clinical and respiratory indicators, such as the severity of respiratory distress, oxygen requirement, and applied airway pressure, our study investigated whether objective and readily available laboratory parameters could provide complementary information. Additional strengths include the use of a well-defined population of preterm infants born at ≤32 weeks of gestation, the selection of surfactant administration within the first 24 h of life as a clinically relevant outcome, the assessment of potential confounders through multivariable analysis, and the comparison of the discriminative performance of continuous variables using ROC curve analysis.

Our study has several limitations. First, its retrospective, single-center design precludes causal inference and limits the generalizability of the findings. In addition, six of the 45 infants in the early surfactant administration group received treatment in the delivery room before collecting the first complete blood count sample. Therefore, the temporal relationship between blood sampling and surfactant administration was not consistent across all patients. This limits our ability to assess the utility of admission complete blood count parameters for identifying infants who may subsequently receive surfactant. Accordingly, our findings should be interpreted as associations between laboratory parameters and surfactant administration within the first 24 h of life, rather than evidence of their ability to predict subsequent treatment.

Delivery-room intubation was more frequent among infants who received early surfactant, potentially reflecting greater initial respiratory disease severity. Because intubation may also directly influence the decision to administer surfactant, its relationship with the outcome is complex. In a sensitivity analysis additionally adjusting for delivery-room intubation, gestational age remained significantly associated with early surfactant administration, whereas the associations of SGA status and the 1 min Apgar score were no longer statistically significant. These findings should be interpreted cautiously because of the reduced sample size, potential residual confounding, and clinical overlap between intubation and surfactant administration. Furthermore, delivery-room respiratory support data were unavailable for six infants because the relevant medical records could not be retrieved or the infants had been referred from other healthcare facilities.

Another important limitation was the incomplete reporting of differential leukocyte counts in the laboratory results. Consequently, NLR, PLR, MLR, and SII could be calculated in only 84 of the 100 infants. The underlying reasons for the missing differential counts could not be established retrospectively. Missing data was disproportionately concentrated among infants who received early surfactant, reducing the effective sample size and potentially limiting statistical power. Furthermore, this unequal distribution raises concerns about possible selection bias. Therefore, the absence of statistically significant associations between these inflammatory indices and early surfactant administration should be interpreted cautiously.

The method of surfactant administration and the use of repeated surfactant doses were not recorded, precluding subgroup analyses according to administration technique and treatment intensity. However, this limitation did not directly affect classification of the primary outcome, defined as receipt of at least one dose of surfactant within the first 24 h of life. Hematological parameters may also be influenced by factors such as blood sampling site, timing of umbilical cord clamping, placental transfusion, and perinatal stress, which could not all be assessed in a standardized manner using retrospective records. Similarly, because the type of blood gas sample and details of respiratory support before sampling were not standardized, the PCO_2_ findings should be interpreted cautiously.

Finally, the relatively small sample size reduced the precision of some association estimates and limited the statistical power of the analyses. Moreover, the ROC-derived cutoff values and the discriminative performance of the multivariable model were evaluated in the development cohort without independent external validation. Therefore, these findings should be considered exploratory and should not be interpreted as evidence of validated predictive performance or immediate clinical applicability. Larger, prospective, multicenter studies with standardized sampling procedures and independent external validation are needed to confirm these associations and assess their potential clinical utility.

## 5. Conclusions

In preterm infants born at ≤32 weeks of gestation, lower gestational age, SGA status, and lower 1 min Apgar scores were associated with early surfactant administration in the primary multivariable analysis. However, only gestational age remained significantly associated with the outcome in the sensitivity analysis that additionally accounted for delivery-room intubation. These findings should therefore be interpreted cautiously, particularly given the uncertainty surrounding the association with SGA status.

Admission hemoglobin and hematocrit levels differed between groups, but hemoglobin was not independently associated with early surfactant administration after adjustment. No statistically significant associations were identified for the evaluated CBC-derived inflammatory indices; however, incomplete laboratory data and the limited sample size restrict the interpretation of these findings.

The observed associations and ROC-derived cutoff values should not be regarded as validated predictors of subsequent surfactant administration. Clinical decisions regarding surfactant therapy should continue to be based on respiratory status, oxygen requirements, and the level of respiratory support. Larger, prospective, multicenter studies with standardized blood sampling before surfactant administration and independent external validation are needed to confirm these associations and evaluate the potential clinical utility of laboratory parameters.

## Figures and Tables

**Table 1:** Demographic and perinatal characteristics of the study population according to early surfactant administration.

Variable	No Early Surfactant Administration (*n* = 55)	Early Surfactant Administration (*n* = 45)	*p*
Gestational age, weeks	31.0 (29.5–32.0)	27.0 (26.0–30.0)	**<0.001**
Birth weight, g	1490 (1107.5–1765)	975 (695–1160)	**<0.001**
Apgar score at 1 min	6 (5–7)	4 (3.25–5)	**<0.001**
Apgar score at 5 min	8 (7–8)	7 (6–8)	**<0.001**
Male sex	31 (56.4)	23 (51.1)	0.747
Birth weight classification			0.140
AGA	44 (80.0)	29 (64.4)	
SGA	7 (12.7)	13 (28.9)	
LGA	4 (7.3)	3 (6.7)	
Cesarean delivery	51 (92.7)	39 (86.7)	0.339
Antenatal corticosteroid exposure ^1^			0.900
One dose	17 (32.1)	12 (30.8)	
Two doses	32 (60.4)	23 (59.0)	
Not administered	4 (7.5)	4 (10.3)	
Delivery room respiratory support ^2^			**0.004**
CPAP	50 (92.6)	27 (67.5)	
Endotracheal intubation	4 (7.4)	13 (32.5)	

Data are presented as median (interquartile range) or n (%). Continuous variables were compared using the Mann–Whitney U test. Categorical variables were compared using Pearson’s chi-square test, Fisher’s exact test, or the Fisher–Freeman–Halton exact test, as appropriate. ^1^ Calculated among the 92 infants with available antenatal corticosteroid data. ^2^ Calculated among the 94 infants with available delivery room respiratory support data. Apgar scores were available for 96 infants. Abbreviations: AGA, appropriate for gestational age; SGA, small for gestational age; LGA, large for gestational age; CPAP, continuous positive airway pressure. Bold values indicate statistically significant results (*p* < 0.05).

**Table 2:** Laboratory parameters according to early surfactant administration.

Variable	No Early Surfactant Administration (*n* = 55)	Early Surfactant Administration (*n* = 45)	*p*
Blood gas parameters			
pH ^1^	7.280 (7.222–7.338)	7.260 (7.203–7.328)	0.708
PCO_2_, mmHg ^2^	48.7 (42.1–55.9)	43.0 (35.8–49.0)	**0.018**
Complete blood count parameters			
WBC, ×10^9^/L	8.86 (6.45–12.55)	7.72 (5.00–11.94)	0.177
Hemoglobin, g/dL	17.73 ± 2.15	15.93 ± 2.95	**0.001**
Hematocrit, %	54.81 ± 6.45	49.87 ± 8.46	**0.002**
Platelet count, ×10^9^/L	243.91 ± 82.28	219.76 ± 82.05	0.147
RDW, %	16.1 (15.0–16.8)	15.3 (14.8–16.7)	0.404
MPV, fL	10.30 ± 0.93	10.58 ± 1.10	0.174
Neutrophil count, ×10^9^/L ^3^	4.27 (2.25–6.46)	2.92 (1.65–5.54)	0.303
Lymphocyte count, ×10^9^/L ^4^	3.96 (2.88–4.80)	3.08 (2.45–4.07)	0.074
Monocyte count, ×10^9^/L ^5^	0.58 (0.26–1.04)	0.57 (0.19–0.96)	0.628
CBC-derived indices			
NLR ^4^	0.92 (0.57–1.58)	0.86 (0.62–1.76)	0.810
PLR ^4^	62.96 (44.31–75.55)	70.91 (52.84–100.45)	0.156
MLR ^4^	0.15 (0.07–0.24)	0.22 (0.04–0.36)	0.458
RPR	0.067 (0.051–0.091)	0.067 (0.058–0.089)	0.390
SII ^4^	204.55 (135.50–376.99)	216.09 (129.39–468.16)	0.732
MPV/platelet ratio	0.045 (0.032–0.055)	0.048 (0.038–0.062)	0.174

Data are presented as mean ± standard deviation or median (interquartile range). Available-case analysis was used for variables with missing data. Student’s *t*-test or the Mann–Whitney U test was used, as appropriate. ^1^ Available in 52 and 44 infants, respectively. ^2^ Available in 53 and 44 infants, respectively. ^3^ Available in 55 and 42 infants, respectively. ^4^ Available in 53 and 31 infants, respectively. ^5^ Available in 54 and 31 infants, respectively. Abbreviations: PCO_2_, partial pressure of carbon dioxide; WBC, white blood cell count; RDW, red cell distribution width; MPV, mean platelet volume; NLR, neutrophil-to-lymphocyte ratio; PLR, platelet-to-lymphocyte ratio; MLR, monocyte-to-lymphocyte ratio; RPR, RDW-to-platelet ratio; SII, systemic immune-inflammation index. Bold values indicate statistically significant results (*p* < 0.05).

**Table 3:** Logistic regression analysis of factors associated with early surfactant administration.

Variable	Univariable OR (95% CI)	*p*	Multivariable OR (95% CI)	*p*
Gestational age, per 1-week increase	0.65 (0.53–0.78)	<0.001	0.65 (0.50–0.84)	**0.001**
SGA	2.79 (1.00–7.74)	0.049	4.61 (1.25–16.91)	**0.021**
Apgar score at 1 min, per 1-point increase	0.50 (0.36–0.70)	<0.001	0.67 (0.46–0.96)	**0.031**
Hemoglobin, per 1 g/dL increase	0.75 (0.63–0.90)	0.002	0.99 (0.78–1.25)	0.911

The multivariable model included gestational age, SGA status, Apgar score at 1 min, and hemoglobin level. The analysis included 96 infants with complete data for all model variables. Birth weight, Apgar score at 5 min, and hematocrit were not entered into the multivariable model because of their strong correlations with gestational age, Apgar score at 1 min, and hemoglobin, respectively. Delivery-room intubation was excluded from the primary model because of its potential temporal and clinical overlap with surfactant administration and was evaluated separately in a sensitivity analysis. Abbreviations: OR, odds ratio; CI, confidence interval; SGA, small for gestational age.

**Table 4:** Receiver operating characteristic (ROC) curve analysis of variables associated with early surfactant administration.

Variable	AUC (95% CI)	Cutoff	Sensitivity, % (95% CI)	Specificity, % (95% CI)	*p*
Gestational age, w	0.776 (0.683–0.870)	≤28	66.7 (51.0–80.0)	81.8 (69.1–90.9)	**<0.001**
Birth weight, g	0.804 (0.715–0.893)	≤1255	82.2 (67.9–92.0)	70.9 (57.1–82.4)	**<0.001**
Hemoglobin, g/dL	0.694 (0.589–0.799)	≤16.2	60.0 (44.3–74.3)	72.7 (59.0–83.9)	**<0.001**
Hematocrit, %	0.677 (0.570–0.784)	≤46.9	40.0 (25.7–55.7)	90.9 (80.0–97.0)	**0.001**
PCO_2_, mmHg	0.640 (0.528–0.751)	≤39.2	40.9 (26.3–56.8)	86.8 (74.7–94.5)	**0.014**

Cutoff values were determined using the Youden index. Values at or below the reported cutoff were associated with early surfactant administration. The 95% confidence intervals for sensitivity and specificity were calculated using the Clopper–Pearson exact binomial method. Because the cutoff values were derived and evaluated in the same cohort, they should be considered exploratory and require external validation before clinical application. Abbreviations: ROC, receiver operating characteristic; AUC, area under the curve; CI, confidence interval; PCO_2_, partial pressure of carbon dioxide.

## Data Availability

The de-identified data supporting the findings of this study are available from the corresponding author upon reasonable request and subject to the relevant institutional and ethical requirements. The data are not publicly available due to patient confidentiality and because the informed consent obtained from the parents or legal guardians did not include unrestricted public sharing of individual-level data.

## References

[1] Huang T.R., Chen H.L., Yang S.T., Su P.C., Chung H.W. (2024). The outcomes of preterm infants with neonatal respiratory distress syndrome treated by minimally invasive surfactant therapy and non-invasive ventilation. Biomedicines.

[2] Sweet D.G., Carnielli V.P., Greisen G., Hallman M., Klebermass-Schrehof K., Lavizzari A., Ozek E., Te Pas A., Roehr C.C., Saugstad O.D. (2026). European Consensus Guidelines on the Management of Respiratory Distress Syndrome: 2025. Neonatology.

[3] Bhandari V., Black R., Gandhi B., Hogue S., Kakkilaya V., Mikhael M., Moya F., Pezzano C., Read P., Roberts K.D. (2023). RDS-NExT workshop: Consensus statements for the use of surfactant in preterm neonates with RDS. J. Perinatol..

[4] Mandy G., White B., Fernandes C.J., Pammi M., Katakam L. (2020). General care (babies <1500 g). Guidelines for Acute Care of the Neonate.

[5] Alkan G., Sert A., Tuzer Oz S.K., Emiroglu M. (2022). Hematological Parameters and Inflammatory Markers in Children with Multisystem Inflammatory Syndrome. Genel Tıp Derg..

[6] Núñez Sanagustín M., Osredkar J. (2026). Blood gas analysis: Clinical applications, interpretation and future directions (Review). Med. Int..

[7] Wolfsberger C.H., Seidel B.N., Fleck L., Herrmann M., Kramer D., Benesch M., Pichler G., Seidel M.G. (2025). Complete blood count reference intervals for extremely preterm neonates. Eur. J. Pediatr..

[8] Fenton T.R., Kim J.H. (2013). A systematic review and meta-analysis to revise the Fenton growth chart for preterm infants. BMC Pediatr..

[9] Özkan H., Erdeve Ö., Kanmaz G., Alan S. (2026). Türk Neonatoloji Derneği Respiratuvar Distres Sendromu ve Surfaktan Tedavi Rehberi 2026. 2. Güncelleme.

[10] Saboute M., Kashaki M., Bordbar A., Khalessi N., Farahani Z. (2015). The incidence of respiratory distress syndrome among preterm infants admitted to neonatal intensive care unit: A retrospective study. Open J. Pediatr..

[11] De Luca D., Autilio C., Pezza L., Shankar-Aguilera S., Tingay D.G., Carnielli V.P. (2021). Personalized Medicine for the Management of RDS in Preterm Neonates. Neonatology.

[12] Malhotra A., Sasi A., Miller S.L., Jenkin G., Polglase G.R. (2014). The efficacy of surfactant replacement therapy in the growth-restricted preterm infant: What is the evidence?. Front Pediatr..

[13] Ng E.H., Shah V. (2021). Guidelines for surfactant replacement therapy in neonates. Paediatr. Child Health.

[14] Brix N., Sellmer A., Jensen M.S., Pedersen L.V., Henriksen T.B. (2014). Predictors for an unsuccessful INtubation-SURfactant-Extubation procedure: A cohort study. BMC Pediatr..

[15] Aslamzai M., Froogh B.A., Mukhlis A.H., Faizi O.A., Sajid S.A., Hakimi Z. (2023). Factors associated with respiratory distress syndrome in preterm neonates admitted to a tertiary hospital in Kabul city: A retrospective cross-sectional study. Glob. Pediatr..

[16] Regassa D.A., Nagaash R.S., Habtu B.F., Haile W.B. (2024). Diagnostic significance of complete blood cell count and hemogram-derived markers for neonatal sepsis at Southwest Public Hospitals, Ethiopia. World J. Clin. Pediatr..

[17] Arcagok B.C., Kandemir I. (2025). Red Cell Distribution Width-to-Platelet Ratio and Other Hematological Markers as Early Predictors of Bronchopulmonary Dysplasia in Preterm Infants. Children.

[18] Cakir U., Tugcu A.U., Tayman C., Yildiz D. (2024). Evaluation of the Effectiveness of Systemic Inflammatory Indices in the Diagnosis of Respiratory Distress Syndrome in Preterm with Gestational Age of ≤32 Weeks. Am. J. Perinatol..

[19] Akın M.S., Akyol O., Okman E., Yazıcı A., Sarı F.N., Alyamaç Dizdar E. (2024). Systemic inflammatory indices as predictors of lung maturation in preterm infants born before 32 weeks of gestation. J. Pediatr. Intensive Care.

[20] Kör A., Çakır U. (2026). Gebelik haftası 32 haftadan küçük prematüre bebeklerde respiratuvar distres sendromu ile ilişkili faktörler. Kırıkkale Üniversitesi Tıp Fakültesi Derg..

